# A PUBLIC HEALTH APPROACH TO BRAIN HEALTH: THE HBI ROAD MAP SERIES

**DOI:** 10.1093/geroni/igad104.0850

**Published:** 2023-12-21

**Authors:** Shelby Roberts, Diane Ty

## Abstract

In 2005, congressional support led to the creation of the Healthy Brain Initiative (HBI) and the longstanding collaboration between the Alzheimer’s Association and the Centers for Disease Control and Prevention to advance understanding of and support for cognitive decline as a central part of public health practice. Since then, HBI partners have worked together to implement public health strategies that promote brain health, address dementia and help support caregivers. The Healthy Brain Initiative Road Map Series guides this effort. The first HBI Road Map was released by CDC and the Alzheimer’s Association in 2007 and was the first framework for viewing cognitive health and dementia as a public health issue. The second HBI Road Map was released in 2013 and expanded the role of state and local public health departments and partners in improving cognitive health. The third HBI Road Map, published in 2018, continued to chart a course for state and local public health departments and their partners by aligning actions with the essential services of public health. The HBI Road Map for Indian Country, published in 2019, is the first public health guide focused on dementia in American Indian and Alaska Native communities. The release of the fourth HBI Road Map in 2023 continues to advance the field and prepare public health departments to better address health equity and build capacity as new treatments become available. This symposium highlights successes of the Road Map Series and the development of the 4th edition of the HBI Road Map.

